# Development and internal validation of a prognostic nomogram for recurrence in chronic rhinosinusitis with nasal polyps after functional endoscopic sinus surgery

**DOI:** 10.3389/fmed.2026.1917341

**Published:** 2026-08-27

**Authors:** Yang Yang, Congli Geng, Jiahua Luo, Min Wang, Xiaopei Yuan, Yan Liu, Shichang Li, Zhimin Xing

**Affiliations:** 1 Department of Otorhinolaryngology, Head and Neck Surgery, Beijing Jishuitan Hospital, Capital Medical University, Beijing, China; 2 Department of Otorhinolaryngology, Head and Neck Surgery, Peking University People’s Hospital, Beijing, China

**Keywords:** nasal polyps, nomogram, recurrence, rhinosinusitis, risk factors

## Abstract

**Objectives:**

Chronic rhinosinusitis with nasal polyps (CRSwNP) frequently recurs after functional endoscopic sinus surgery (FESS). This study aimed to develop and internally validate a prognostic nomogram for predicting the two-year recurrence-free rate (RFR) in patients with CRSwNP after FESS.

**Methods:**

A total of 404 patients with CRSwNP who underwent FESS were retrospectively enrolled and randomly divided into a training cohort (n = 303) and an internal validation cohort (n = 101). Recurrence was defined as the first postoperative detection of nasal polyps by nasal endoscopy within 2 years. Candidate predictors were selected using LASSO Cox regression and further evaluated using multivariate Cox regression. A nomogram was constructed to predict the two-year RFR.

**Results:**

During the two-year follow-up, 148 patients experienced recurrence. Five independent predictors were included in the final model: number of previous nasal surgeries, olfactory cleft polyp, Lund–Mackay score, peripheral blood eosinophil percentage, and tissue eosinophil percentage. The nomogram showed good discrimination, with C-indices of 0.751 (95% CI, 0.707–0.795) in the training cohort and 0.707 (95% CI, 0.625–0.789) in the internal validation cohort. Calibration curves demonstrated good agreement between predicted and observed two-year RFR. Risk stratification based on the nomogram score separated patients into low-, intermediate-, and high-risk groups with significantly different RFRs.

**Conclusion:**

We developed and internally validated a nomogram for estimating two-year recurrence-free rate in patients with CRSwNP after FESS. The nomogram will help identify patients at high risk of recurrence and assist in the development of individualized treatment plans for patients with CRSwNP. Furthermore, we found that olfactory cleft polyps were a new independent risk factor for the postoperative recurrence of CRSwNP.

## Introduction

Chronic rhinosinusitis (CRS) is an inflammatory condition characterized by specific nasal symptoms that last for > 12 weeks; the diagnosis can be confirmed based on endoscopic and/or computed tomography (CT) findings (1, 2). CRS is clinically classified into CRS with nasal polyps (CRSwNP) or CRS without nasal polyps (CRSsNP). CRSwNP affects approximately 1.1–4.3% of the general population (3, 4). Despite adequate medical treatment and surgical intervention, CRSwNP has a high recurrence rate of 21.8–62% (5–11). Long-term medication and multiple surgical treatments cause huge economic and medical burdens for patients with recurrent disease and for the society (12, 13). Knowledge of the risk factors for CRSwNP recurrence will help clinicians in preparing individualized and appropriate treatment plans for patients with CRSwNP. However, predicting the recurrence of CRSwNP remains challenging.

Several risk factors have been reported to be correlated with CRSwNP recurrence, including gender, asthma, overweight, dust exposure, endotype, prior endoscopic sinus surgery (ESS), acetylsalicylic acid (ASA) intolerance, blood eosinophil count, serum total immunoglobulin E (IgE) level, blood basophil count, tissue eosinophil infiltration, preoperative polyposis severity, Lund–Mackay CT score, CT ethmoid (E) score ≥ maxillary (M) score, CT E/M ratio, and biological parameters (6–9, 14–36). Different studies have reported different risk factors. Further studies are required to integrate multiple independent risk factors to achieve an accurate and visual prediction of CRSwNP prognosis. A nomogram is a novel model that integrates multiple risk factors to predict the prognosis.

In this study, we aimed to identify predictors of postoperative recurrence in patients with CRSwNP and to develop and internally validate a nomogram for estimating the two-year recurrence-free rate after FESS. We also explored the prognostic value of olfactory cleft polyps, a variable that has not been fully incorporated into previous prediction models.

## Materials and methods

### Patients

The present study was approved by the Institutional Review Board of Peking University People’s Hospital, and the written informed consent was provided by the participants. CRSwNP was diagnosed according to the European Position Paper on Rhinosinusitis and Nasal Polyps (EPOS) 2020 guidelines (1). From January 2014 to December 2019, a total of 404 CRSwNP patients were enrolled in this study, with an average age of 46.8 ± 14 years; the ratio of men-to-women was 2.2:1. Patients were followed up for 24 months after functional endoscopic sinus surgery (FESS). All patients were randomly assigned to either a training (n = 303) cohort and an internal validation cohort (n = 101) at a ratio of 3:1 using a computer-generated random allocation procedure. All patients met the following inclusion criteria: (1) diagnosed with CRSwNP and underwent FESS; (2) patients who did not receive corticosteroids or antibiotics within the four-week period before surgery, or patients with asthma who did not receive systemic corticosteroids, or patients who did not receive targeted biologic therapy; (3) patients with complete clinical data, including nasal endoscopy, CT, and pathological data. The exclusion criteria were as follows: (1) patients with tumours, fungal rhinosinusitis, choanal polyps, haemorrhagic necrotizing polyps, sinus haemorrhage, or blood disease; (2) patients lost to follow-up within 2 years of surgery.

### Variables analysed

Demographic and clinical data were collected from medical records, including information on sex, age, comorbidities, family history of nasal polyps, previous nasal surgeries, clinical symptoms and signs, nasal endoscopy, CT, peripheral blood results, and histopathological findings. Allergic disease was diagnosed based on the symptoms and results of intradermal skin testing and serum total IgE testing for common allergens using a fluoroenzyme immunoassay. Serum total IgE level ≥ 0.35 kU/L was defined as a positive result. Asthma was diagnosed on the basis of previous pulmonary function test results. The inhaled allergens included mugwort, *Ambrosia*, *Artemisia*, *Humulus*, dust mites (*Dermatophagoides pteronyssinus* and *D. farinae*), cockroaches, cat hair, dog hair, *Aspergillus*, *Penicillium notatum*, *Cladosporium*, and *Alternaria*. Before surgery, nasal endoscopic examinations were conducted by an independent observer, and CT images were evaluated by two independent radiologists. The ratio of the ethmoid (E) sinus score to the maxillary (M) sinus score was defined as the E/M ratio, as previously described (19). Polypoid changes in the nasal cavity were confirmed by nasal endoscopic examination and surgery. Blood samples were obtained before surgery and analysed using an automatic analyser in the same laboratory. Polyp tissues for histopathologic testing were obtained during surgery, sectioned into 4-μm thick slices, and stained with haematoxylin and eosin (H&E). The numbers of eosinophils and total inflammatory cells in the tissues were counted at high-power (HP) magnification (× 400) using a light microscope, and 10 HP fields (HPFs) were randomly selected. The tissue eosinophil percentage was calculated as the eosinophil count per HPF/number of total inflammatory cells per HPF. Eosinophilic CRSwNP (ECRSwNP) was defined as the presence of more than 10 eosinophils per HPF, as recommended by the EPOS 2020 guidelines (1).

### Treatment and follow-up

All patients underwent FESS performed by experienced rhinologists. The extent of surgery was determined according to the distribution and severity of disease on preoperative nasal endoscopy and CT imaging. The procedures followed standard FESS principles with the aim of complete removal of visible polyps and restoration of sinus ventilation and drainage. Because the enrolled patients underwent surgery between 2014 and 2019, postoperative medical treatment after FESS followed the EPOS 2012 recommendations that were used in clinical practice during that period, including long-term (at least 12 weeks) intranasal corticosteroids, short-term (7–21 days) systemic corticosteroids, and at least 14 days of broad-spectrum or culture-directed antibiotics (37). The postoperative follow-up schedules were as follows: 2 weeks, 1 month, 3 months, 6 months, 1 year, and 2 years after FESS. Disease recurrence was defined as the first postoperative detection of nasal polyps by nasal endoscopy during the scheduled follow-up period after FESS. Follow-up endoscopic examinations were performed by experienced rhinologists according to routine clinical practice. Because of the retrospective nature of the study, the evaluators were not blinded to clinical information. The time of last follow-up of lost patients was recorded.

### Statistical analysis

The statistical software SPSS (version 23.0; IBM SPSS, Armonk, NY, USA) and R software (version 4.0.5; Vienna, Austria), including the packages ‘rms,’ ‘survival,’ ‘survminer,’ and ‘glmnet,’ were used. Student’s *t*-test or Mann–Whitney *U* test was used for continuous variables according to the data distribution. Categorical variables were analysed using the χ^2^ test or Fisher’s exact test. Two-sided *p* < 0.05 was considered statistically significant.

Candidate predictors were selected based on clinical relevance and previously reported associations with postoperative recurrence of CRSwNP. Categorical variables were coded as binary indicators, and continuous variables were entered as continuous predictors. Patients with missing data in key predictors or follow-up outcomes were excluded before model development. Univariate Cox regression and LASSO Cox regression were performed in the training cohort for variable selection. Continuous variables were standardized before LASSO analysis, and lambda.1se was selected by 10-fold cross-validation to obtain a parsimonious model. Variables with nonzero coefficients at lambda.1se were included in the multivariate Cox proportional hazards regression model to identify independent predictors and calculate hazard ratios (HRs) with 95% confidence intervals (CIs). The proportional hazards assumption was evaluated using Schoenfeld residuals. The multivariate Cox proportional hazards model of predicted 2-year RFR was: 2-year RFR = 0.653 ^ exp. (0.407 × number of previous nasal surgery + 0.654 × olfactory cleft polyp + 0.062 × Lund–Mackay score + 0.096 × blood eosinophil percentage + 0.014 × tissue eosinophil percentage), where olfactory cleft polyp was coded as 0 for absence and 1 for presence. A nomogram based on the final multivariate Cox model was constructed to predict the two-year recurrence-free probability in patients with CRSwNP after FESS. Model performance was evaluated using Harrell’s C-index and calibration plots with 1,000 bootstrap resamples. Risk stratification was performed according to cutoff values of the total nomogram score determined by X-tile analysis in the training cohort, and then applied to the internal validation cohort. Kaplan–Meier curves, log-rank tests, and decision curve analysis were used to assess the clinical utility of the model.

## Results

### Patient characteristics

**Figure 1:**
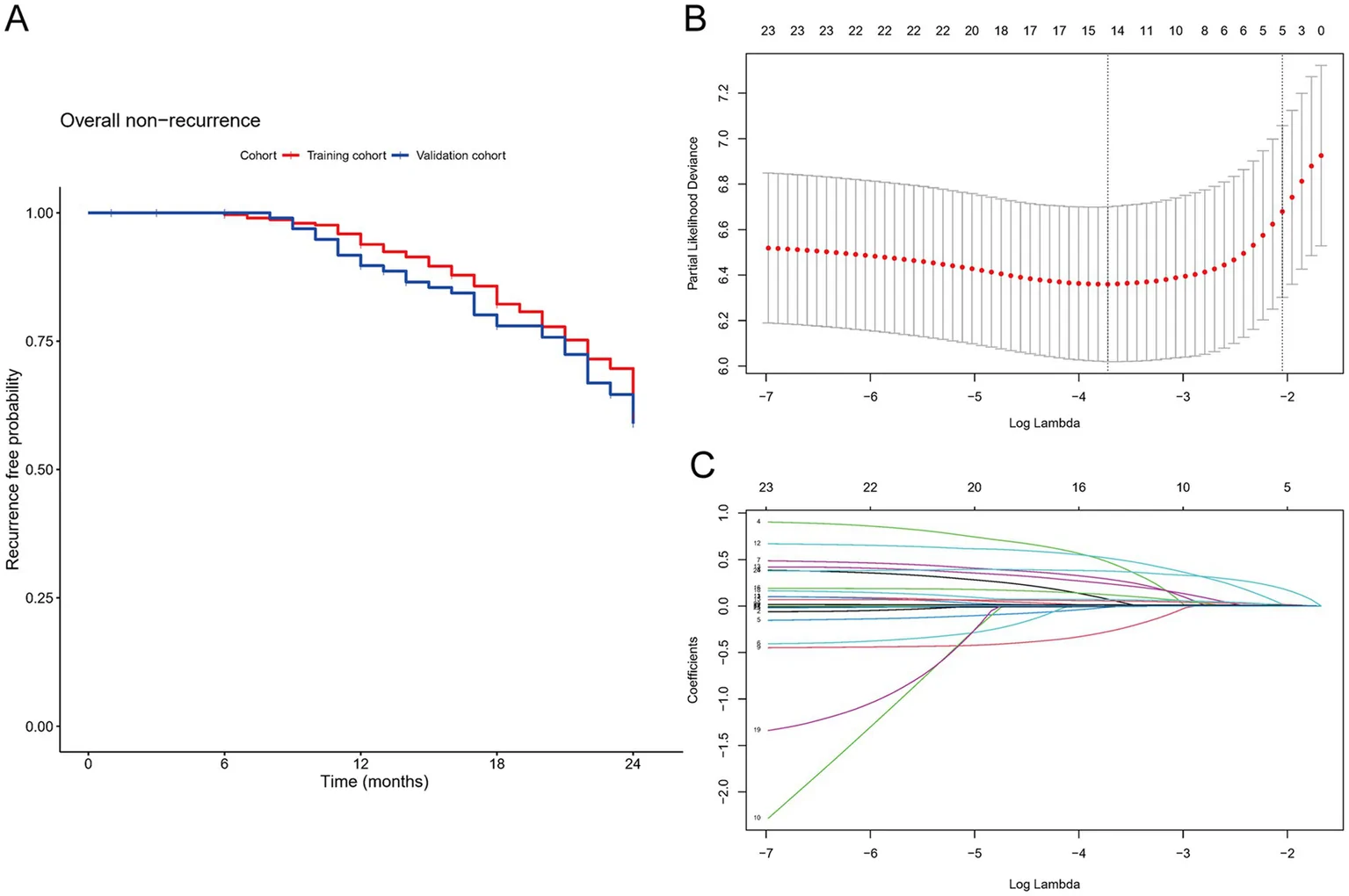
Kaplan–Meier survival curves of the recurrence free rate in training and validation cohorts **(A)**, and the 10-fold cross-validation for optimal variables selection in the LASSO model **(B)**, and the LASSO coefficient profile of 23 variables **(C)**.

The demographic and clinical characteristics of the training and validation cohorts are presented in Table 1. No significant differences were observed in the distribution of patients between the two cohorts (*p* > 0.05) according to age, sex, asthma, allergic rhinitis, ASA intolerance, positive result for inhaled allergen test, frequency of nasal symptoms, or two-year recurrence rate. The Kaplan–Meier survival curves showed no significant difference in the RFRs between the two cohorts (log-rank test; *p* = 0.615) (Figure 1).

**Table 1:** Patient baseline characteristics by cohort.

Characteristics	Total (*n* = 404)	Training cohort (*n* = 303)	Validation cohort (*n* = 101)	*p* value*
Age (mean ± *SD*, years)	46.8 ± 14.0	46.7 ± 14.1	47.5 ± 13.5	0.606
Sex, n (%)				0.291
Male	279 (69.1)	205 (67.7)	74 (73.3)	
Female	125 (30.9)	98 (32.3)	27 (26.7)	
Asthma, n (%)				0.862
Yes	225 (55.7)	168 (55.4)	57 (56.4)	
No	179 (44.3)	135 (44.6)	44 (43.6)	
Allergic rhinitis, n (%)				0.784
Yes	92 (22.8)	68 (22.4)	24 (23.8)	
No	312 (77.2)	235 (77.6)	77 (76.2)	
ASA intolerance, n (%)				0.745
Yes	13 (3.2)	9 (3.0)	4 (4.0)	
No	391 (96.8)	294 (97.0)	97 (96.0)	
Positive inhaled allergen test, n (%)				0.67
Yes	135 (33.4)	103 (34.0)	32 (31.7)	
No	269 (66.6)	200 (66.0)	69 (68.3)	
Symptoms				
Nasal obstruction, n (%)				0.164
Yes	386 (95.5)	292 (96.4)	94 (93.1)	
No	18 (4.5)	11 (3.6)	7 (6.9)	
Headache/facial pain, n (%)				0.07
Yes	123 (30.4)	85 (28.1)	38 (37.6)	
No	281 (69.5)	218 (71.9)	63 (62.4)	
Hyposmia/anosmia, n (%)				0.165
Yes	327 (80.9)	250 (82.5)	77 (76.2)	
No	77 (19.1)	53 (17.5)	24 (23.8)	
Runny nose, n (%)				0.24
Yes	328 (81.2)	250 (82.5)	78 (77.2)	
No	76 (18.8)	53 (17.5)	23 (22.8)	
Two-year recurrence rate, n (%)				0.812
Yes	148 (36.6)	110 (36.3)	38 (37.6)	
No	256 (63.4)	193 (63.7)	63 (62.4)	

CRSwNP, chronic rhinosinusitis with nasal polyp. Comparison between training cohort and validation cohort.

### Independent prognostic factors for CRSwNP recurrence in the training dataset

The univariate analysis revealed that factors such as allergic rhinitis (HR = 1.683; *p* = 0.012), number of previous nasal surgeries (HR = 1.699; *p* < 0.001), hyposmia/anosmia (HR = 2.657; *p* = 0.005), olfactory cleft polyp (HR = 2.516; *p* < 0.001), Lund–Kennedy endoscopic score (HR = 1.129; *p* = 0.003), polypoid change of the middle turbinate (HR = 1.601; *p* = 0.017), Lund–Mackay CT score (HR = 1.108; *p* < 0.001), E/M ratio (HR = 1.36; *p* = 0.001), peripheral blood eosinophil percentage (HR = 1.131; *p* < 0.001), peripheral blood eosinophil absolute count (HR = 4.179; *p* < 0.001), tissue eosinophil percentage (HR = 1.022; *p* < 0.001), tissue eosinophil absolute count (HR = 1.005; *p* < 0.001), and ECRSwNP (HR = 3.529; *p* < 0.001) were significantly different between the groups showing recurrence or non-recurrence of CRSwNP in the training cohort (Table 2). In the LASSO model, five optimal variables with nonzero coefficients were identified: number of previous nasal surgeries, olfactory cleft polyps, Lund–Mackay score, peripheral blood eosinophil percentage, and tissue eosinophil percentage (Figure 1). These five variables met the criteria for proportional hazards assumption. The multivariate Cox regression analysis revealed that five independent risk factors, including number of previous nasal surgeries (HR = 1.503; *p* < 0.001), olfactory cleft polyp (HR = 1.924; *p* = 0.003), Lund–Mackay score (HR = 1.063; *p* = 0.003), peripheral blood eosinophil percentage (HR = 1.1; *p* = 0.001), and tissue eosinophil percentage (HR = 1.014; *p* = 0.001), were significantly associated with CRSwNP recurrence (Table 3).

**Table 2:** Univariate Cox regression analysis for two-year recurrence in the training cohort.

Characteristics	Univariate Cox analysis		
	*β*	HR (95% *CI*)	*p* value
Age, years	0.000	1.000 (0.987–1.013)	0.96
Sex (male/female)	0.162	1.175 (0.791–1.746)	0.423
Asthma (absence/ presence)	0.362	1.437 (0.978–2.111)	0.065
Allergic rhinitis (absence/ presence)	0.52	1.683 (1.119–2.531)	**0.012**
ASA intolerance (absence/ presence)	0.716	2.046 (0.834–5.020)	0.118
Number of previous nasal surgery	0.53	1.699 (1.408–2.049)	**<0.001**
Positive inhaled allergen	0.187	1.206 (0.802–1.813)	0.368
Nasal obstruction (absence/ presence)	0.259	1.296 (0.441–4.082)	0.658
Headache/facial pain (absence/ presence)	0.268	1.308 (0.878–1.948)	0.186
Hyposmia/anosmia (absence/ presence)	0.977	2.657 (1.343–5.255)	**0.005**
Runny nose (absence/ presence)	−0.263	0.769 (0.485–1.217)	0.262
Olfactory cleft polyp (absence/ presence)	0.923	2.516 (1.638–3.867)	**<0.001**
Lund-Kennedy score	0.121	1.129 (1.041–1.224)	**0.003**
Polypoid change of middle turbinate (absence/ presence)	0.47	1.601 (1.089–2.352)	**0.017**
Lund-Mackay score	0.103	1.108(1.065–1.153)	**<0.001**
E/M ratio	0.308	1.360 (1.129–1.638)	**0.001**
Serum total IgE (kU/L)	0.000	1.000 (0.999–1.001)	0.683
Blood eosinophil percentage	0.123	1.131 (1.076–1.189)	**<0.001**
Blood eosinophil count (cells × 10^9/L)	1.43	4.179 (2.069–8.441)	**<0.001**
Tissue eosinophil percentage	0.021	1.022 (1.014–1.030)	**<0.001**
Tissue eosinophil count (cells/HPF)	0.005	1.005 (1.003–1.007)	**<0.001**
Tissue inflammatory cell count (cells/HPF)	0.002	1.002 (1.000–1.003)	0.06
ECRSwNP (tissue eosinophil count > 10 cells/HPF)	1.261	3.529 (1.936–6.431)	**<0.001**

β, β-coefficients; HR, hazard ratio; CI, confidence interval; ASA, acetylsalicylic acid; E/M ratio, ratio of computed tomography score for ethmoid sinus and maxillary sinus; HPF, high power field; ECRSwNP, eosinophilic chronic rhinosinusitis with nasal polyps.

**Table 3:** Multivariate Cox regression analysis for two-year recurrence in the training cohort.

Characteristics	Multivariate Cox analysis		
	*β*	HR (95% *CI*)	*p* value
Number of previous nasal surgery	0.407	1.503 (1.240–1.822)	**<0.001**
Olfactory cleft polyp (absence/ presence)	0.654	1.924 (1.243–2.978)	**0.003**
Lund-Mackay score	0.062	1.063 (1.021–1.107)	**0.003**
Blood eosinophil percentage	0.096	1.100 (1.039–1.166)	**0.001**
Tissue eosinophil percentage	0.014	1.014 (1.006–1.023)	**<0.001**

β, β-coefficients; HR, hazard ratio; CI, confidence interval.

### Constructing and validating a prognostic nomogram

**Figure 2:**
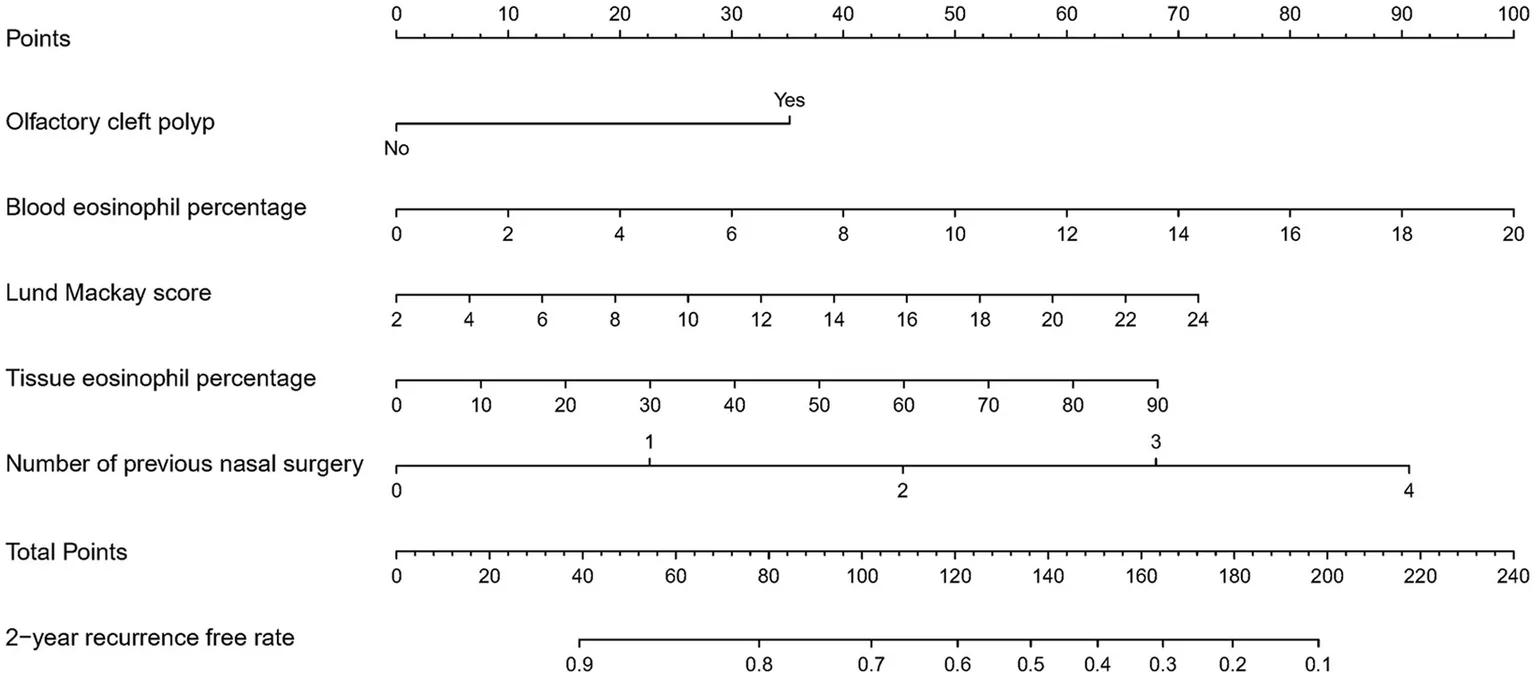
Predictive nomogram for estimating the two-year recurrence-free rate in patients with CRSwNP after functional endoscopic sinus surgery. A higher total score indicates a lower predicted two-year recurrence-free rate.

**Figure 3:**
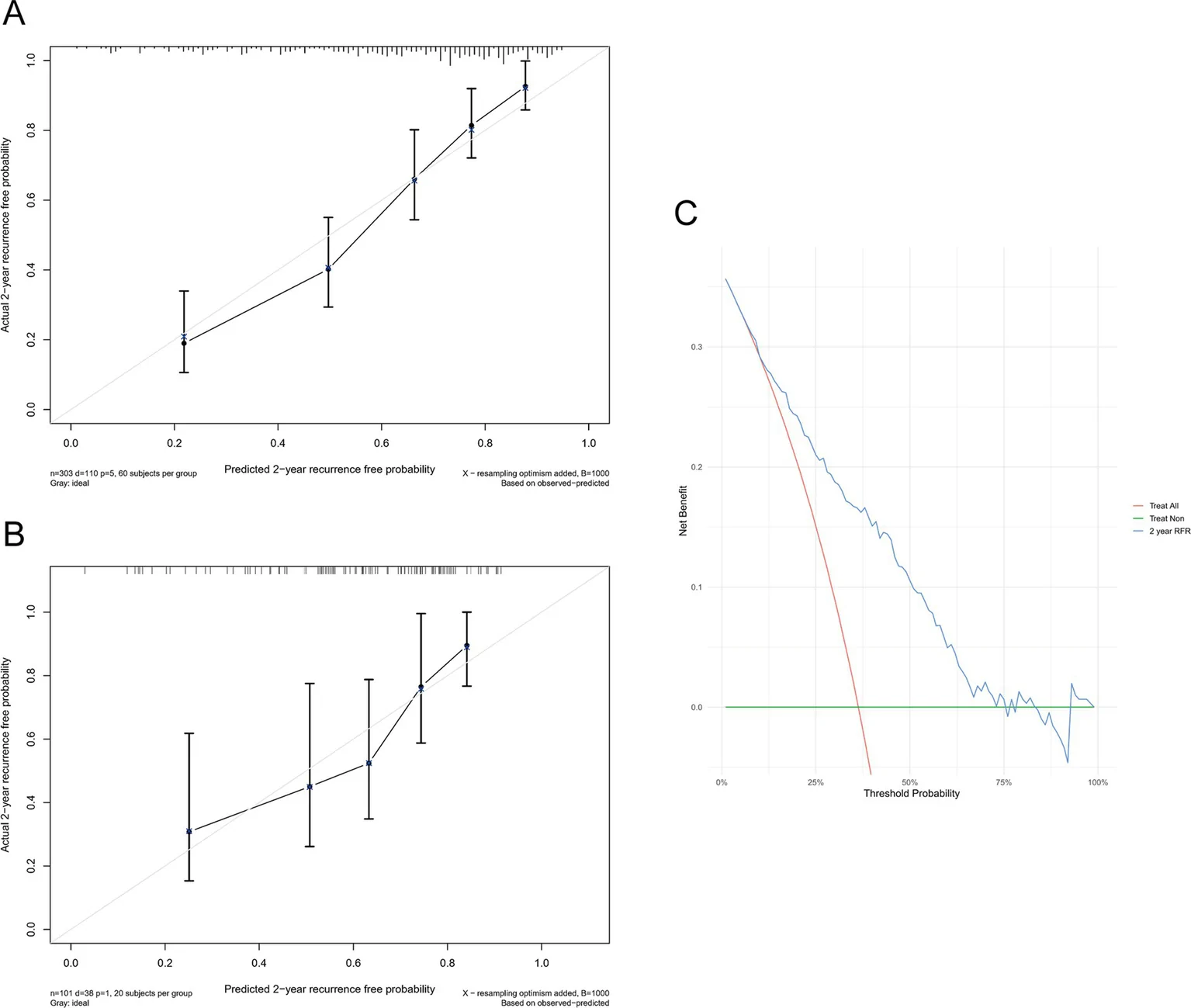
Calibration plot of the nomogram in the training **(A)** and validation **(B)** cohorts. Decision curve analysis of nomogram achieved a higher net benefit than the treat-all or treat-none strategy **(C)**.

Based on the results of the multivariate Cox regression analysis, the number of previous nasal surgeries, olfactory cleft polyps, Lund–Mackay score, peripheral blood eosinophil percentage, and tissue eosinophil percentage were selected for the construction of the final nomogram model. The nomogram for two-year prognosis of the training cohort is shown in Figure 2. A higher total score indicated a higher risk of recurrence and a lower predicted two-year RFR. The C-indices of the nomogram were 0.751 (95% confidence interval [CI], 0.707–0.795) and 0.707 (95% CI, 0.625–0.789) for the training and validation cohorts, respectively, indicating that the nomogram had good discriminative ability. Two calibration plots were prepared to validate the constructed nomogram in the training and validation cohorts using 1,000 repetitions of bootstrap sample corrections. The predicted RFR showed a good correlation with the actual observation. Decision curve analysis demonstrated that the nomogram achieved a higher net benefit than the treat-all or treat-none strategy across a clinically relevant range of threshold probabilities, suggesting potential clinical utility for individualized postoperative risk assessment (Figure 3).

### Kaplan–Meier curves for RFR after stratifying according to risk classification

**Figure 4:**
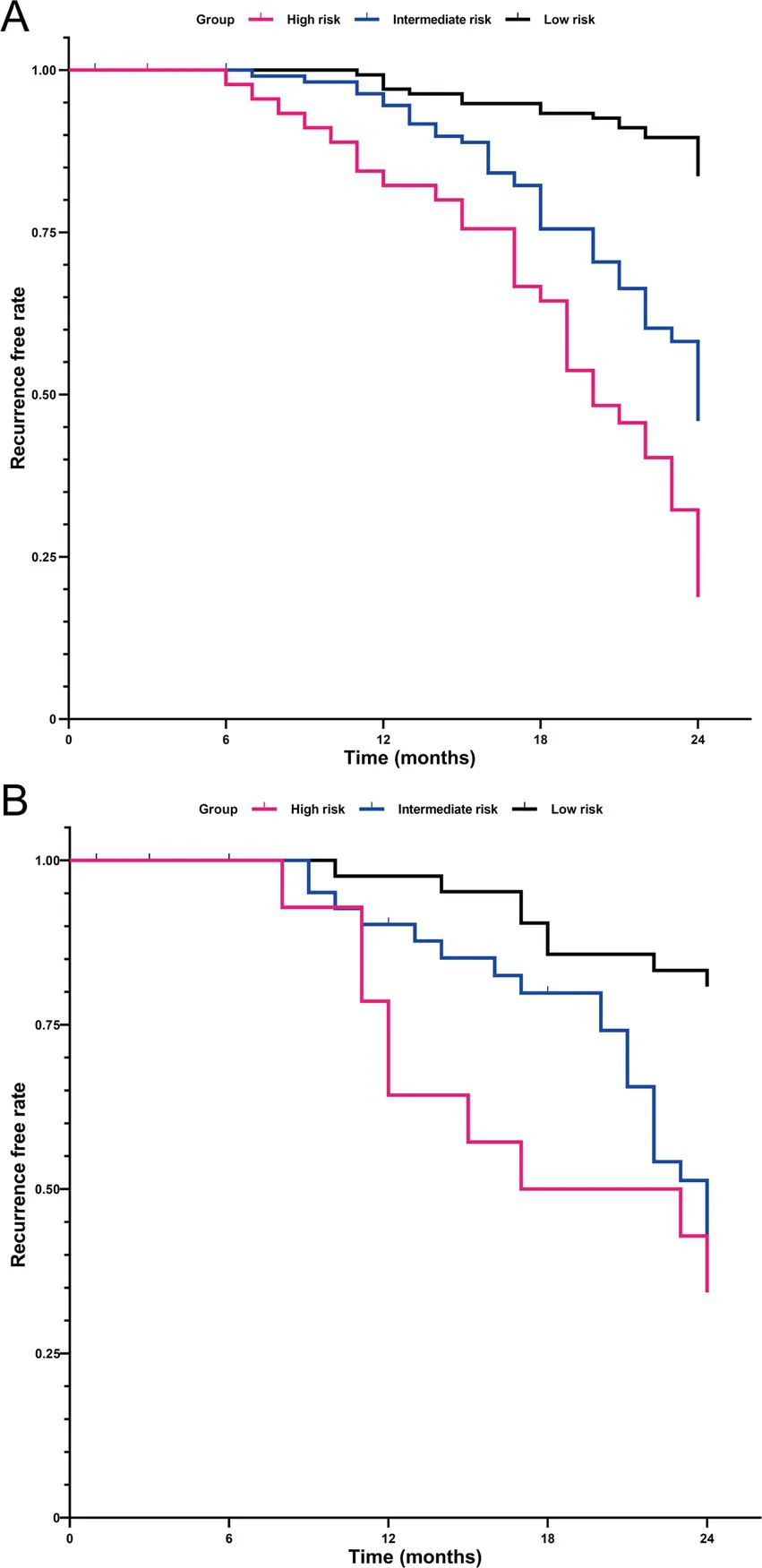
Kaplan–Meier curves for recurrence-free rate (RFR) after stratifying according to risk classification using the training **(A)** and validation **(B)** cohorts.

Risk stratification was performed using the total nomogram score. The optimal cutoff values were determined in the training cohort using X-tile software, yielding two cutoff points of 105 and 164. Patients were therefore classified into low-risk (<105), intermediate-risk (105–164), and high-risk (>164) groups (Table 4). These prespecified cutoffs derived from the training cohort were subsequently applied to the validation cohort. The Kaplan–Meier survival curves for the training and validation cohorts showed significant differences among the low-, intermediate-, and high-risk groups (*p* < 0.001) (Figure 4).

**Table 4:** Risk stratification according to the nomogram total score.

Recurrence risk group	Total score range	Predicted two-year recurrence free rate	Training cohort (n = 303)	Validation cohort(n = 101)
High risk	>164	<0.30	46	14
Intermediate risk	105–164	0.30–0.68	113	41
Low risk	<105	>0.68	144	46

## Discussion

In this study, we developed a new nomogram by combining LASSO with Cox regression analysis to predict the RFR in patients with CRSwNP after FESS. Five factors were integrated in the nomogram: olfactory cleft polyps, number of previous nasal surgeries, Lund–Mackay score, peripheral blood eosinophil percentage, and tissue eosinophil percentage. The prognostic nomogram based on these risk factors provided an intuitive and user-friendly recurrence scoring system compared with that obtained using other prognostic models.

In this study, olfactory cleft polyps were present in up to 57.9% of the patients with CRSwNP. Olfactory cleft polyps originate from the midline of the nasal cavity, including the middle turbinate, superior turbinate, and posterior upper part of the nasal septum. Meng et al. (19) found that loss of smell was more severe in patients with CRSwNP recurrence than that in patients without CRSwNP recurrence. Olfactory cleft opacification, observed by performing sinus CT scans, was increased in patients with aspirin-exacerbated respiratory disease (AERD) compared to that in patients without AERD (38). Eosinophilic chronic rhinosinusitis (ECRS) is a subtype of CRS with a high recurrence rate. Ethmoid-dominated changes observed during CT scans were an important factor and were assessed to determine the severity of ECRS (39). Polypoid changes in the central sinonasal compartment are associated with inhalant allergies (40). Studies have not been conducted to analyse the relationship between olfactory cleft polyps and the prognosis of patients with CRSwNP. In this study, the presence of olfactory cleft polyps was an independent risk factor for the recurrence of nasal polyps after FESS in patients with CRSwNP (HR, 1.924; 95% CI, 1.243–2.978). Olfactory cleft polyps may not only be used as indicators of ECRS but can also be used to predict CRSwNP recurrence.

Consistent with the findings of previous studies, our results revealed that the number of previous nasal surgeries was associated with postoperative nasal polyp recurrence (7, 18). Moreover, time to failure after previous surgery was also associated with recurrence rates, and a time interval of 3 years or less from previous surgery was a good predictor of recurrence (41). In this study, all patients with CRSwNP underwent FESS to avoid disease recurrence caused by incomplete lesion removal. Jankowski et al. (42) found that radical ethmoidectomy was more effective than functional ethmoidectomy in reducing nasal polyp recurrence. Both Draff III frontal sinus surgery and middle turbinate resection result in low recurrence rates of nasal polyps (43, 44). We should investigate whether the recurrence of nasal polyps can be effectively prevented by performing extensive surgeries in patients who are at a high risk of recurrence.

The results revealed that the sinus CT Lund–Mackay total scores and the E/M score ratios were related to the recurrence of nasal polyps. This finding is consistent with those of previous studies (45, 46). Tao et al. (22) reported that a sinus CT Lund–Mackay score ≥ 15 and CT ethmoid sinus score ≥ maxillary sinus score were independent risk factors for uncontrolled CRS. Meng et al. (19) proposed that a preoperative sinus CT E/M score ratio > 2.55 had a high predictive value for CRSwNP recurrence. In the present study, however, only the sinus CT Lund–Mackay total score was retained as an optimal predictor in the LASSO regression analysis, whereas the E/M ratio was excluded. This does not necessarily indicate that the E/M ratio is biologically irrelevant. Rather, LASSO penalization prioritizes predictors that provide independent incremental predictive value in the presence of correlated variables. Therefore, the predictive information conveyed by the E/M ratio may partly overlap with the Lund–Mackay score.

In many studies, tissue eosinophilia has been found to be a meaningful prognostic risk factor for nasal polyp recurrence. Brescia et al. (17) reported that histological evidence of ECRSwNP was an independent prognostic indicator of nasal polyp recurrence. Nakayama et al. (20) observed that mucosal eosinophilia (> 70 eosinophils/HPF) was significantly associated with poor prognosis in Japanese patients with CRSwNP. In a European cohort study, the threshold for mucosal eosinophilia was set to 120 eosinophils/HPF because it predicted a high risk of recurrence (47). Zhong et al. reported that the tissue eosinophil percentage was one of risk factors for postoperative recurrence in CRSwNP (27). Similar to the findings of previous studies, our results revealed that the tissue eosinophil percentage, tissue eosinophil count, and ECRSwNP type were significantly higher in the group showing recurrence of CRSwNP compared to that in the group without recurrence of CRSwNP. However, tissue eosinophil percentage, not tissue eosinophil count, remains an optimal risk factor. The tissue eosinophil percentage is a better predictor of the risk of CRSwNP recurrence than the tissue eosinophil count.

The blood eosinophil count can be a reliable marker for predicting CRS recurrence. Several studies have suggested that blood eosinophil counts are positively correlated with the risk of CRS recurrence and may be reduced post-ESS (14, 24, 48). However, the cut-off value of blood eosinophil counts for recurrence prediction remains controversial. Brescia et al. (24) reported that a high blood eosinophil percentage (≥ 5.9%) predicts a high risk of recurrence. A blood eosinophil ratio of > 2.5% was used to predict uncontrolled postoperative CRS (22). An advantage of the nomogram developed in this study is that because the nomogram was not based on a cut-off value of blood eosinophil counts, it can predict the recurrence risks associated with different blood eosinophil percentages.

There are some researches aiming to development nomogram models for the prediction of recurrence after endoscopic sinus surgery in patients with CRSwNP, while the included risk factors and the predictive capabilities of the models vary (16, 49–52). Kirtsreesakul et al. (50) developed one nomogram model for long-term recurrence risk prediction in patients with CRSwNP following ESS, showing strong discrimination, with area under the ROC curve (AUROC) of 0.878, 0.870, 0.886, and 0.873 at 2, 5, 10, and 15 years post-ESS. Du et al. reported one nomogram model consisting of blood eosinophils, total serum IgE level, asthma, and the number of previous ESSs, demonstrating a C index of 0.81 and 0.80 for predicting recurrence in the training and validation cohorts (16). Our study was the first to incorporate the olfactory cleft polyp as a risk factor. Moreover, it presented the first nomogram capable of stratifying the risk of postoperative recurrence within 2 years. This model demonstrated robust predictive performance in an internal validation cohort.

This study has several limitations. First, its retrospective design may have introduced selection bias. Second, although the nomogram was internally validated using a random split-sample approach, external validation in independent cohorts is still required. Third, recurrence was defined as the first endoscopic detection of nasal polyps, which may not fully reflect clinically significant outcomes such as uncontrolled disease, systemic corticosteroid use, revision surgery, or biologic initiation. Postoperative medication adherence was also not systematically recorded. Fourth, the prediction horizon was limited to 2 years, precluding assessment of long-term recurrence risk. Finally, none of the patients received biologic therapy before surgery; therefore, the nomogram may not be applicable to biologic-treated populations. Future prospective studies with standardized outcome assessment, longer follow-up, and inclusion of biologic exposure are warranted.

Physicians need to identify patients with CRSwNP who are at a high risk of recurrence after FESS at an early stage and subsequently formulate individual treatment plans and follow-up schedules. We developed and internally validated a reliable and user-friendly nomogram model for predicting the prognosis of patients with CRSwNP. This nomogram could be used to develop specific management strategies for patients with CRSwNP.

## Data Availability

The raw data supporting the conclusions of this article will be made available by the authors, without undue reservation.
